# Benzofuranyl Esters: Synthesis, Crystal Structure Determination, Antimicrobial and Antioxidant Activities

**DOI:** 10.3390/molecules200916566

**Published:** 2015-09-11

**Authors:** C. S. Chidan Kumar, Li Yee Then, Tze Shyang Chia, Siddegowda Chandraju, Yip-Foo Win, Shaida Fariza Sulaiman, Nurul Shafiqah Hashim, Kheng Leong Ooi, Ching Kheng Quah, Hoong-Kun Fun

**Affiliations:** 1 X-ray Crystallography Unit, School of Physics, Universiti Sains Malaysia, Penang 11800, Malaysia; E-Mails: lyeeth0923@hotmail.com (L.Y.T.); chiatzeshyang@hotmail.com (T.S.C.); hkfun@usm.my or hfun.c@ksu.edu.sa (H.-K.F.); 2 Department of Engineering Chemistry, Alva’s Institute of Engineering & Technology, Visvesvaraya Technological University, Mijar, Moodbidri 574225, Karnataka, India; 3 Department of Sugar Technology & Chemistry, University of Mysore, Sir M. V. PG Center, Tubinakere, Mandya 571402, Karnataka, India; E-Mail: chandraju1@yahoo.com; 4 Department of Chemical Science, Faculty of Science, Universiti Tunku Abdul Rahman, Perak Campus, Jalan Universiti, Bandar Barat, Kampar 31900, Malaysia; E-Mail: yipfw@utar.edu.my; 5 School of Biological Sciences, Universiti Sains Malaysia, Penang 11800, Malaysia; E-Mails: shaida@usm.my (S.F.S.); nshafiqah25@gmail.com (N.S.H.); ooilng@yahoo.com (K.L.O.); 6 Department of Pharmaceutical Chemistry, College of Pharmacy, King Saud University, Riyadh 11451, Saudi Arabia

**Keywords:** benzofuran, spectroscopic analysis, antimicrobial, antioxidant, XRD, substituent

## Abstract

A series of five new 2‐(1‐benzofuran‐2‐yl)‐2‐oxoethyl 4-(un/substituted)benzoates **4**(**a**–**e**), with the general formula of C_8_H_5_O(C=O)CH_2_O(C=O)C_6_H_4_X, X = H, Cl, CH_3_, OCH_3_ or NO_2_, was synthesized in high purity and good yield under mild conditions. The synthesized products **4**(**a**–**e**) were characterized by FTIR, ^1^H-, ^13^C- and ^1^H-^13^C HMQC NMR spectroscopic analysis and their 3D structures were confirmed by single-crystal X-ray diffraction studies. These compounds were screened for their antimicrobial and antioxidant activities. The tested compounds showed antimicrobial ability in the order of **4b** < **4a** < **4c** < **4d** < **4e** and the highest potency with minimum inhibition concentration (MIC) value of 125 µg/mL was observed for **4e**. The results of antioxidant activities revealed the highest activity for compound **4e** (32.62% ± 1.34%) in diphenyl-2-picrylhydrazyl (DPPH) radical scavenging, **4d** (31.01% ± 4.35%) in ferric reducing antioxidant power (FRAP) assay and **4a** (27.11% ± 1.06%) in metal chelating (MC) activity.

## 1. Introduction

Benzo[*b*]furan nucleus is widespread in plants and often the natural products possessing benzofuran are useful for their immense pharmacological properties. Systematic investigation of this heterocyclic compound plays important roles in the development of medicinal chemistry and synthetic products [1,2]. Pyromucic acid (2-furoic acid) is the first prepared furan compound discovered in 1780 [3]. Basically, benzo[*b*]furan ring systems bearing various substituents at the C-2 position are broadly distributed and had been reported to possess antifungal, antiviral and antioxidant activities [4,5]. Some 2-arylbenzofuran derivatives are well-known biodynamic agents possessing a wide range of biological activities, including calcium blockers, phytoestrogens, antioxidative, anticancer, insecticidal, antiproliferative, antiviral, antifungal, antiplatelet, anti-inflammatory, immunosuppressive, antifeedant and cancer preventative activity [6,7,8,9,10,11,12,13,14,15,16,17,18]. Besides the bioactivities, benzo[*b*]furan derivatives can act as building blocks for fluorescent sensors [19] and optical brighteners.

Encouraged by the biological activities associated with the benzo[*b*]furan ring system, we herein report the efficient synthesis, spectra, X-ray crystal structure analysis and biological activities of five new benzo[*b*]furan esters **4**(**a**–**e**).

## 2. Results and Discussion

### 2.1. Chemistry

Generally, the compounds **4**(**a**–**e**) revealed absorption bands above 3000 cm^−1^ which indicate the presence of unsaturated C-H (benzene and benzofuran) groups whereas the methyl group, -CH_3_, as well as methylene group, -CH_2_- revealed asymmetric and symmetric C-H stretching frequencies around 2966–2858 cm^−1^ [20]. The infrared spectra studies of **4**(**a**–**e**) also revealed the presence of ν(C=C) bands which are usually found for benzene and benzofuran groups. In addition, the compounds **4**(**a**–**e**) revealed two distinct ν(C=O) bands in the range of 1725–1682 cm^−1^ in which the ν(C=O) band with the lower wave number is attributed to the C=O of carboxylate anion [21,22,23,24]. Two exceptional cases which are the presence of ν(C-Cl) at 1086 cm^−1^ in **4b** and the presence of ν(NO_2_) at 1524 cm^−1^ in **4e** were observed.

The ^1^H-NMR spectra of compounds **4**(**a**–**e**) exhibited similarities among each other with the presence of -CH_2_- protons centering around δ ≈ 5.55 ppm and two sets of well-resolved doublet centering around δ ≈ 7.31 and 8.09 ppm with the integration values of 2:2, ascribed to the -CH- protons of benzene group [20]. Compound **4e** revealed one exceptional sharp singlet peak at 8.32 ppm with the integration value of 4 indicating the total number of benzene protons. This peak is originated from the attached NO_2_ group at the *para* position of benzene ring. In addition, all the five protons of benzofuran are located in the downfield region in the ^1^H-NMR spectra centering around δ ≈ 7.33, 7.51, 7.57, 7.63 and 7.73 ppm with the integration values of 1:1:1:1:1. The exceptional and predictable observations are the occurrence of -CH_3_ and -OCH_3_ proton signals of compounds **4c** and **4d** in the upfield region at 2.42 and 3.87 ppm, respectively. Based on the integration values, the number of protons in compounds **4**(**a**–**e**) are in accordance with the number of protons proposed.

All compounds exhibited three distinct sets of carbon signals in the ^13^C-NMR spectra. In the downfield region of ^13^C NMR spectra, both δ(C=O) and δ(COO) signals are located at δ ≈ 183.71 ppm and δ ≈ 165.47 ppm, respectively, whereas the -CH_2_- carbon signals are located in the upfield region centering around δ ≈ 66.42 ppm [22,23,24]. The exceptional and predictable observations are the occurrence of -CH_3_ and -OCH_3_ carbon signals of compounds **4c** and **4d**, respectively, in the upfield region of ^13^C-NMR spectra. In the ^13^C-NMR spectra study, the carbon signals of benzene and benzofuran groups were found in the range of 112.59–155.79 ppm [21,22,23,24]. The carbon signals centering at δ ≈ 112.60, 113.60, 123.62, 124.33, 126.98, 128.87, 150.63 and 155.79 ppm are attributed to benzofuran groups and the remaining signals are attributed to benzene carbons.

### 2.2. X-ray Crystal Structure Description

2-(Benzofuran-2-yl)-2-oxoethyl 4-(un/substituted)benzoates with different functional groups *viz.*, no substitution (**4a**), -chloro (**4b**), -methyl (**4c**), -methoxy (**4d**) and -nitro (**4e**) substituted at the -*para* position of phenyl ring were subjected to X-ray diffraction analysis. Crystal data and refinement parameters of the analyzed compounds are listed in Table 1. The hydrogen bonds geometry and π∙∙∙π interactions are presented in Table S1 and Table S2 (Supplementary Materials).

**Table 1:** Crystal data and refinement parameters for **4**(**a**–**e**).

Compound	4a	4b	4c	4d	4e
CCDC Deposition Number	1,037,756	1,037,759	1,037,762	1,037,763	1,037,764
Molecular Formula	C_17_H_12_O_4_	C_17_H_11_ClO_4_	C_18_H_14_O_4_	C_18_H_14_O_5_	C_17_H_11_NO_6_
Molecular Weight	280.27	314.71	294.29	310.29	325.27
Crystal System	Monoclinic	Monoclinic	Monoclinic	Triclinic	Triclinic
Space Group	*P*2_1_/*n*	*C*2/*c*	*P*2_1_/*n*	$P\bar{1}$	$P\bar{1}$
*a* (Å)	10.2230(9)	33.483(5)	12.5266(13)	6.9335(6)	6.5424(10)
*b* (Å)	8.4353(7)	5.3687(8)	6.7689(7)	8.4724(7)	13.197(2)
*c* (Å)	16.1205(15)	26.404(4)	18.0335(19)	12.8702(11)	16.973(3)
α (°)	90	90	90	103.539(2)	79.694(3)
β (°)	98.300(2)	114.833(3)	108.165(2)	95.789(2)	87.211(3)
γ (°)	90	90	90	92.664(2)	85.715(3)
*V* (Å^3^)	1375.6(2)	4307.6(10)	1452.9(3)	729.36(11)	1436.9(4)
*Z*	4	12	4	2	4
*D*_calc_ (g·cm^−3^)	1.353	1.456	1.345	1.413	1.504
Crystal Dimension (mm)	0.25 × 0.36 × 0.48	0.08 × 0.17 × 0.46	0.14 × 0.24 × 0.46	0.20 × 0.27 × 0.41	0.12 × 0.20 × 0.41
µ (mm^−1^)	0.10	0.28	0.10	0.10	0.12
*T*_min_/*T*_max_	0.901/0.976	0.857/0.977	0.903/0.987	0.899/0.980	0.903/0.986
Reflections Measured	15,170	17,994	16,236	14,866	37,178
Indices Range (*h*, *k*, *l*)	−14, 9;	−40, 40;	−17, 16;	−9, 9;	−7, 7;
	−9, 11;	−6, 6;	−9, 9;	−11, 11;	−15, 15;
	−22, 22	−32, 32	−25, 24	−18, 18	−20, 20
θ Limit (°)	2.2–30.2	1.7–26.0	1.8–30.0	1.6–30.2	1.6–25.0
Unique Reflections	4037	4235	4234	4283	4946
Observed Reflections (*I* > 2σ(*I*))	2630	2044	2835	3078	3889
Parameters	190	397	200	209	433
Goodness of Fit on *F*^2^	1.04	0.98	1.03	1.07	1.11
*R*_1_,*wR*_2_ [*I* > 2σ(*I*)]	0.047, 0.162	0.044, 0.143	0.045, 0.148	0.045, 0.149	0.065, 0.216

**Figure 1:**
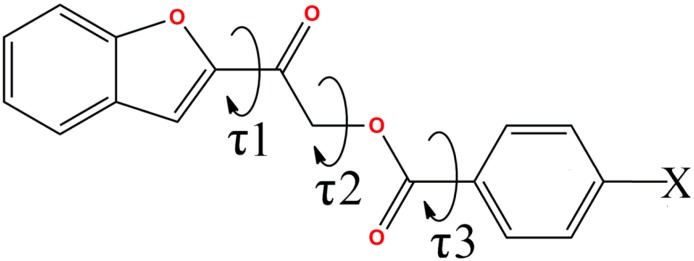
General chemical diagram of **4**(**a**–**e**) shows τ1, τ2 and τ3 torsion angles.

Molecular conformations of these compounds are characterized by three degree-of-freedom, which are the O1–C8–C9–O3, C9–C10–O2–C11 and O2–C11–C12–C13 torsion angles or denoted hereafter as τ1, τ2 and τ3, respectively (Figure 1). Basically, torsion angles τ1 and τ3 are close to 0° or ±180°, indicating small deviations of the ethanone and carboxylate groups from attached benzofuran and phenyl rings, respectively. Since these deviations are very small, the 1-(benzofuran-2-yl)ethanone group and benzoate group are taken as two rigid bodies.

**Figure 2:**
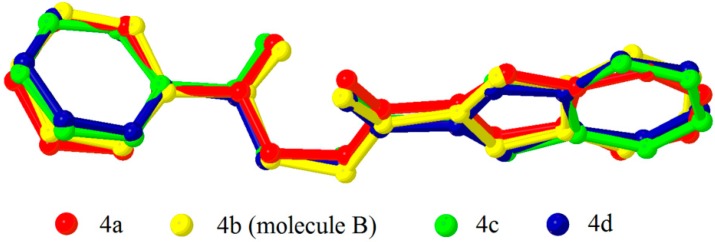
Overlay diagram of **4a**, **4b** (inverted molecule B), **4c** and **4d** shows similar, nearly-perpendicular conformation.

**Figure 3:**
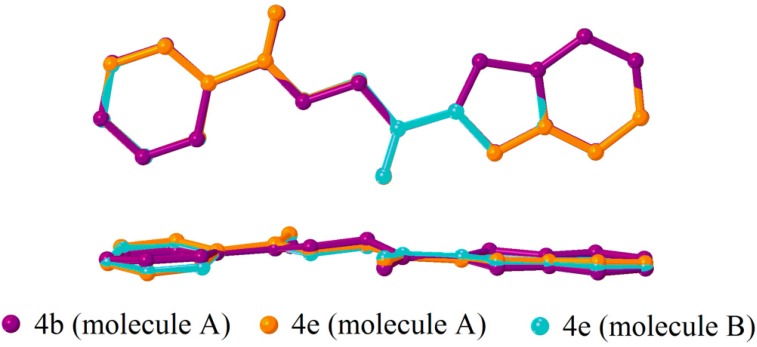
Overlay diagram of nearly flat conformer, **4b** (molecule A), **4e** (molecules A and B), viewed (up) perpendicular to or (down) along the mean plane.

The asymmetric units of compounds **4a**, **4c** and **4d** consist of one unique molecule, while two crystallographically independent molecules (molecules A and B) are observed in compounds **4b** and **4e**. Basically, the compounds under study tend to adopt two types of conformations, in which the benzofuran group is either nearly-planar or nearly-perpendicular with respect to the phenyl ring, as indicated by the torsion angle τ2. Compounds **4a**, **4c** and **4d** adopt nearly-perpendicular conformation with τ2 close to 90°, **4e** is a nearly-planar conformer with τ2 approaches 180° and interestingly, **4b** exhibits both in its crystal structure as summarized in Table 2. The overlay diagrams of both conformers are depicted in Figure 2 and Figure 3. Overall, the values of τ2 are still in good agreement with the conformation distribution of phenacyl benzoate derivatives reported earlier [25] after the replacement of phenyl ring with benzofuran ring.

**Table 2:** Torsion angles τ1, τ2 and τ3 for **4**(**a**–**e**).

Compound	O1–C8–C9–O3 (τ1,°)	C9–C10–O2–C11 (τ2,°)	O2–C11–C12–C13 (τ3,°)
**4a**	−176.92(14)	75.19(17)	171.53(13)
**4b**	5.3(4), −175(2)	162.5(2), −70.2(13)	−176.7(2), −172(2)
**4c**	−3.5(2)	78.36(16)	−171.25(12)
**4d**	−2.62(19)	79.49(15)	−169.34(11)
**4e**	7.2(4), 2.5(4)	177.4(2), 179.6(3)	−175.4(3), −175.6(3)

**Figure 4:**
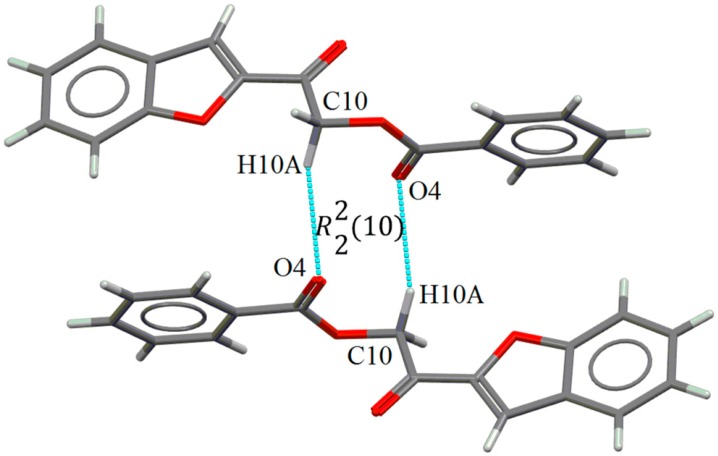
Intermolecular C–H∙∙∙O hydrogen bonds (blue color) form dimeric structures in **4a**.

**Figure 5:**
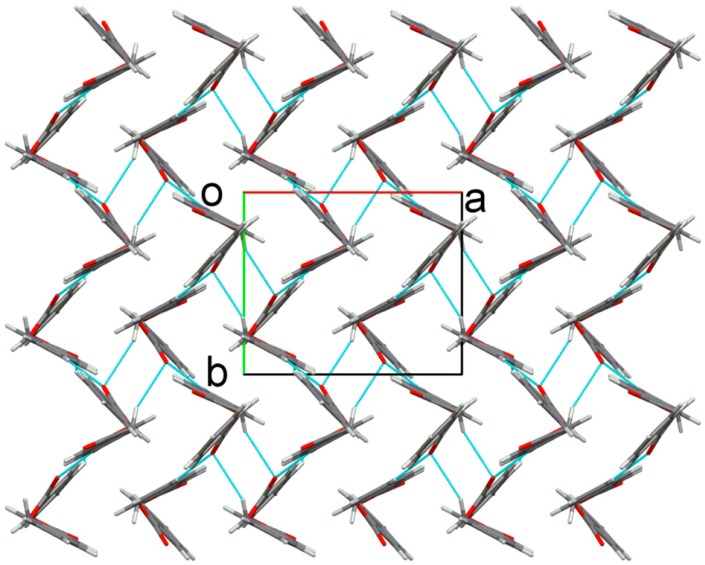
Three-dimensional assembly in **4a** with hydrogen bonds (blue dotted lines).

In compound **4a**, molecules are linked by weak intermolecular C10–H10*A*∙∙∙O4 hydrogen bonds, involving the methylene and carbonyl groups, into zero-dimensional dimeric structures (Figure 4) with $R_{2}^{2}$(10) graph-set motif. This hydrogen-bonded structure is also observed in other related structures such as 2-oxo-2-phenylethyl benzoate [26], 2-(4-bromophenyl)-2-oxoethyl 2-aminobenzoate [25] and 2-(4-bromophenyl)-2-oxoethyl 4-methoxybenzoate [27], suggesting this dimeric form is a favoured interaction pattern in compound with nearly-perpendicular conformation. Weak intermolecular C4–H4*A*∙∙∙O4 and C14–H14*A*∙∙∙O3 hydrogen bonds (Table S1, Supplementary Materials) further connect the dimers into a three-dimensional network (Figure 5).

**Figure 6:**
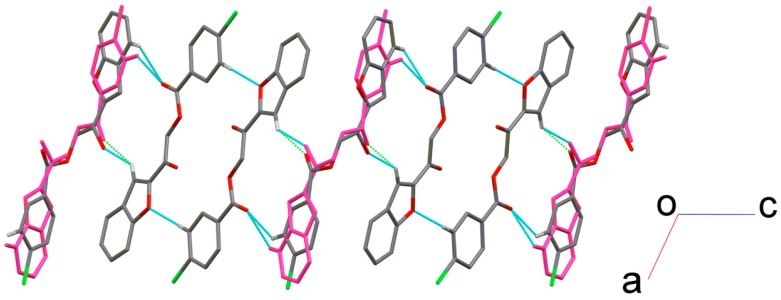
Hydrogen-bonded infinite chain in **4b**. Blue dotted lines are weak C–H∙∙∙O hydrogen bonds. The minor component of disordered molecule B is shown in pink.

The asymmetric part of compound **4b** consists of two independent molecules (A and B). Molecule A adopts nearly-flat conformation whereas molecule B adopts nearly-perpendicular conformation (Table 1, Figure 2 and Figure 3). Molecule B is treated as whole-molecule disorder with 0.5:0.5 site occupancies ratio in which the major and minor components are related with a two-fold rotation symmetry. In the crystal, weak intermolecular C–H∙∙∙O hydrogen bond links the molecules into one-dimensional infinite zigzag chains along *c*-axis (Figure 6).

**Figure 7:**
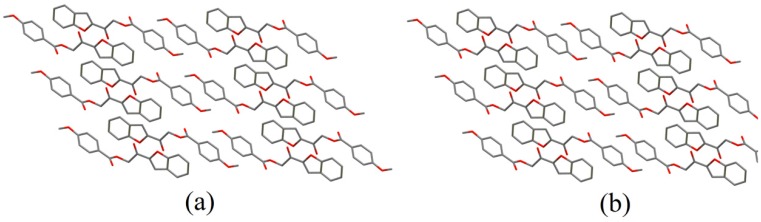
Two-dimensional structural similarities in (**a**) **4c** and (**b**) **4d**.

Compounds **4c** and **4d** are two nearly-perpendicular conformers which show two-dimensional structural similarity (Figure 7). In **4c**, molecules are linked by weak C5–H5*A*∙∙∙O1 hydrogen bond into infinite chains, propagating along the crystallographic *b*-axis. In **4d**, molecules are joined by three weak intermolecular C4–H4*A*∙∙∙O4, C5–H5*A*∙∙∙O3 and C18–H18*A*∙∙∙O5 hydrogen bonds (Table S1, Supplementary Materials) into sheets parallel to (011) plane.

In compound **4e**, the asymmetric unit consists of two independent molecules (A and B) and both adopt nearly-planar conformation with maximum deviations of 0.343 Å and 0.275 Å from their respective mean planes. The molecules are linked by weak C–H∙∙∙O hydrogen bonds (Table S1, Supplementary Materials) and π∙∙∙π interactions into a three-dimensional network. The π∙∙∙π interactions in **4e** (Table S2, Supplementary Materials) are the most redundant among all current compounds **4**(**a**–**e**) due to the layered structure. Additional information on C–H∙∙∙π and π∙∙∙π interactions can be found in Supplementary Materials.

### 2.3. Antimicrobial Activities of the Synthesized Compound

The minimum inhibition concentration (MIC) values of compounds **4**(**a**–**e**) against eight different pathogenic microorganisms are given in Table 3. Compounds **4d** with -methoxy and **4e** with -nitro substitutions were found to inhibit all the tested microorganisms. Compound **4e** gave the lowest MIC value (125 µg/mL) against the Gram-negative bacterium *Salmonella typhimurium* ATCC 14028, Gram-positive bacterium *Streptococcus mutans* ATCC 25175 and the yeast *Candida albicans* ATCC 10231. The MIC value of this compound against other bacteria was found to be 250 µg/mL. Compound **4d** showed MIC value of 250 µg/mL against one Gram-negative bacterium (*Salmonella typhimurium*), all the tested Gram-positive bacteria and the yeast, whereas MIC value of 500 µg/mL was observed against the other Gram-negative bacteria. The MIC value for **4c** was consistent (500 µg/mL) against all tested microorganisms excluding *Bacillus licheniformis*. This bacterium was found to be resistant against **4c**. No activity was found for **4b** against all the tested microorganisms and this result may be due to the -chloro substitution. In short, the results revealed the increase in antimicrobial activity follows with the substitution of -methyl, -methoxy and -nitro, respectively, at the 4-position of the parental skeleton.

**Table 3:** Minimum inhibition concentration (MIC) of the synthesized compounds.

Compound	Gram Negative				Gram Positive			Yeast
	*Escherichia coli* ATCC 25922	*Klebsiella pneumoniae* ATCC 13883	*Pseudomonas aeruginosa* ATCC 27853	*Salmonella typhimurium* ATCC 14028	*Bacillus licheniformis* ATCC 12759	*Streptococcus mutans* ATCC 25175	*Staphylococcus aureus* ATCC 700699	*Candida albicans* ATCC 10231
**4a**	1000	1000	1000	1000	1000	1000	1000	1000
**4b**	-	-	-	-	-	-	-	-
**4c**	500	500	500	500	-	500	500	500
**4d**	500	500	500	250	250	250	250	250
**4e**	250	250	250	125	250	125	250	125
Tetracycline	0.977	1.953	7.813	0.977	1.953	0.244	31.25	0.122

### 2.4. Antioxidant Activities of the Synthesized Compound

The antioxidant capacities were systematically assessed using three different assays at an initial concentration of 8 mg/mL (final concentration of 2 mg/mL). The scavenger capacity was determined by measuring the decrease in absorption of diphenyl-2-picrylhydrazyl (DPPH) radicals. Meanwhile, the reducing power was measured by ferric reducing antioxidant power (FRAP) method to observe the reduction of ferric tripyridyltriazine (Fe(III)-TPTZ) complex to ferrous (Fe(II)-TPTZ) at low pH. These two assays are mainly used to measure the direct involvement of the compounds in enhancing the primary antioxidant activity, whereas the metal chelating assay measures the indirect ability of compounds to act as secondary antioxidant by binding to ferrous (Fe(II)) ion catalyzing oxidation and disrupting the formation of Fe(II)-ferrozine complex.

**Figure 8:**
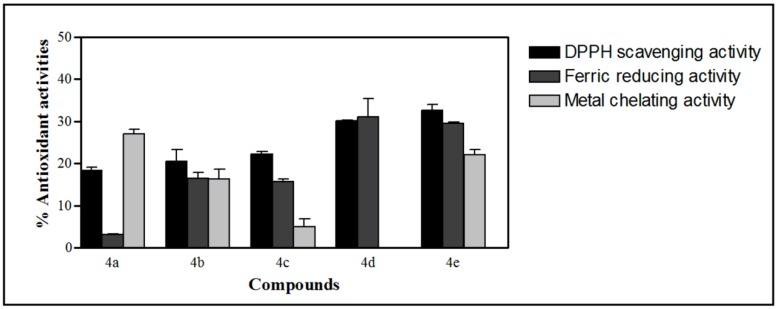
DPPH scavenging, ferric reducing and metal chelating activities of compounds **4**(**a**–**e**) at final concentration of 2 mg/mL. Each value represents the mean ± SD of triplicate analyses.

In general, low antioxidant activities were observed for the tested compounds which might be related to the absence of hydroxyl moiety in the compounds. The results obtained from these assays revealed the highest activity for compound **4e** (32.62% ± 1.34%) in DPPH assay (Figure 8), compound **4d** (31.01% ± 4.35%) in ferric reducing activity and compound **4a** (27.11% ± 1.06%) in metal chelating activity.

## 3. Experimental Section

### 3.1. Instrumentation

The infrared spectra were recorded using a Perkin-Elmer System 2000 FTIR Spectrophotometer as KBr disc in the frequency range of 4000–400 cm^−1^. The spectra for ^1^H-, ^13^C- and ^1^H-^13^C HMQC NMR were recorded on a JEOL JNM-ECX 400 FT-NMR Spectrometer using deuterated CDCl_3_ as the solvent and tetramethylsilane, TMS as the internal standard.

### 3.2. X-ray Diffraction Analysis

Bruker APEX II DUO CCD area-detector diffractometer was used to perform X-ray analysis on these five colourless samples. MoKα radiation (λ = 0.71073 Å) was applied and φ and ω scans were employed in the data collection. The raw data was first reduced using SAINT and absorption correction process was carried out later by SADABS program. In this analysis, all the crystallographic data were collected at room temperature. The SHELXTL [28] program was used to solve the structure with direct methods. Refinements of the structures on *F*^2^ were done using full-matrix least-squares techniques. Anisotropic refinements were applied on all non-hydrogen atoms. All C-bound hydrogen atoms were calculated geometrically with the isotropic displacement parameters set to 1.2 (or 1.5 for methyl group) times the equivalent isotropic U values of the parent carbon atoms. The Olex^2^ [29] software was used to sketch the overlay diagrams. Crystallographic data for compounds **4(a**–**e)** have been deposited at the Cambridge Crystallographic Data Centre with CCDC deposition numbers of 1037756, 1037759 and 1037762-1037764 respectively. These data can be obtained free of charge via http://www.ccdc.cam.ac.uk/conts/retrieving.html (or from the CCDC, 12 Union Road, Cambridge CB2 1EZ, UK; Fax: +44 1223 336033; E-mail: deposit@ccdc.cam.ac.uk).

### 3.3. Synthesis

#### 3.3.1. Synthesis of 1-(Benzofuran-2-yl)ethanone (**2**) and -(Benzofuran-2-yl)-2-bromoethan-1-one (**3**)

Salicylaldehyde (**1**) (0.1 mol), chloroacetone (0.1 mol) and anhydrous potassium carbonate (30 g) were dissolved in dry acetone (150 mL) and the mixture was refluxed for about 12 h. After the mixture was cooled, the filtrate was removed under reduced pressure to obtain a yellow crude product of 1-(benzofuran-2-yl)ethanone (**2**) [30]. The crude product was recrystallized from petroleum ether and purity of the product were checked by using TLC plate with silica gel and acetone:benzene (1:1) solvent system. Next, **2** was refluxed with NBS and petroleum ether in methanol at 333 K for about 2 h. After the reaction completed, the mixture was allowed to cool and filtered. The resultant crude product was dried and recrystallized from ethanol to obtain **3** [25].

#### 3.3.2. General Procedure for the Synthesis of 2‐(1‐Benzofuran‐2‐yl)‐2‐oxoethyl 4 (Unsubstituted/substituted)benzoates **4**(**a**–**e**)

**Figure 9:**
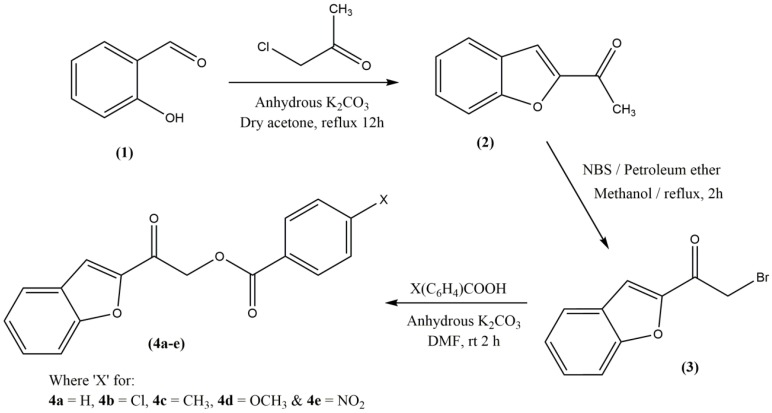
Reaction scheme for the synthesis of target compounds **4**(**a**–**e**).

1-(Benzofuran-2-yl)-2-bromoethan-1-one (**3**) (0.5 mol), substituted benzoic acid (0.6 mol) and anhydrous potassium carbonate (0.5 g) were dissolved in dimethyl formamide (8 mL) and the mixture was then stirred at room temperature for about 2 h. The progress of the reaction was monitored by TLC. After the completion of the reaction, the mixture was poured into a 100 mL beaker containing crushed ice. The precipitate formed was filtered, dried and recrystallized from acetone to get pure products of **4**(**a**–**e**) (Figure 9) [25,31,32].

*2-(1-Benzofuran-2-yl)-2-oxoethyl benzoate* (**4a**): Solvent for crystal growth: Acetone; m.p.: 126–128 °C; Yield: 78%; FTIR as KBr disc (cm^−1^): ν(C-H) aromatic 3124, 3062; ν(C-H) aliphatic 2953, ν(C=O) 1725, 1698; ν(C=C) 1559, 1475; ν(C-O) 1276, 1259. ^1^H-NMR (ppm) (CDCl_3_): δ: benzofuran protons 7.33 (t, 7.8 Hz, 1H); 7.48 (t, 7.8 Hz, 2H overlap with benzene proton); 7.52 (dd, 4.4 Hz, 1.4 Hz, 1H); 7.63 (s, 1H); 7.73 (d, 8.3 Hz, 1H); benzene protons 7.59 (d, 8.7 Hz, 2H); 8.15 (d, 8.2 Hz, 2H); CH_2_ 5.55 (s, 2H). ^13^C-NMR (ppm) (CDCl_3_): δ: benzofuran carbons 112.60, 113.59, 123.61, 124.30, 126.80, 128.84, 150.56, 155.74; benzene carbons 128.59, 129.29, 130.13, 133.58; CH_2_ 66.36, COO 166.05, C=O 183.84.

*2‐(1‐Benzofuran‐2‐yl)‐2‐oxoethyl 4‐chlorobenzoate* (**4b**): Solvent for crystal growth: Acetone; m.P.: 116–118 °C; Yield: 82%; FTIR as KBr disc (cm^−1^): ν(C-H) aromatic 3124, 3093, 3072; ν(C-H) aliphatic 2941, 2914; ν(C=O) 1723, 1697; ν(C=C) 1558, 1486; ν(C-O) 1271, ν(C-Cl) 1086. ^1^H-NMR (ppm) (CDCl_3_): δ: benzofuran protons 7.34 (t, 7.8 Hz, 1H); 7.51 (td, 6.9 Hz, 0.9 Hz, 1H); 7.58 (d, 8.2 Hz, 1H); 7.63 (s, 1H); 7.72 (d, 7.8 Hz, 1H); benzene protons 7.44 (d, 8.7 Hz, 2H); 8.07 (d, 8.2 Hz, 2H); CH_2_ 5.54 (s, 2H). ^13^C-NMR (ppm) (CDCl_3_): δ: benzofuran carbons 112.60, 113.61, 123.63, 124.34, 127.75, 128.90, 150.49, 155.75; benzene carbons 126.77, 128.96, 131.51, 140.07; CH_2_ 66.46, COO 165.22, C=O 183.59.

*2‐(1-Benzofuran‐2‐yl)‐2‐oxoethyl 4‐methylbenzoate* (**4c**): Solvent for crystal growth: Acetone; m.p.: 154–156 °C; Yield: 79%; FTIR as KBr disc (cm^−1^): ν(C-H) aromatic 3115, 3098; ν(C-H) aliphatic 2932, ν(C=O) 1721, 1682; ν(C=C) 1552, 1476; ν(C-O) 1269. ^1^H-NMR (ppm) (CDCl_3_): δ: benzofuran protons 7.33 (t, 7.3 Hz, 1H); 7.51 (t, 6.9 Hz, 0.9 Hz, 1H); 7.59 (d, 7.8 Hz, 1H); 7.62 (s, 1H); 7.72 (d, 7.8 Hz, 1H); benzene protons 7.27 (d, 8.2 Hz, 2H); 8.03 (d, 8.2 Hz, 2H); CH_3_ 2.42 (s, 3H); CH_2_ 5.52 (s, 2H). ^13^C-NMR (ppm) (CDCl_3_): δ: benzofuran carbons 112.59, 113.54, 123.59, 124.27, 126.82, 128.79, 150.60, 155.74; benzene carbons 126.55, 129.31, 130.16, 144.34; CH_3_ 21.85, CH_2_ 66.24, COO 166.09, C=O 183.99.

*2‐(1‐Benzofuran‐2‐yl)‐2‐oxoethyl 4‐methoxybenzoate* (**4d**): Solvent for crystal growth: Acetone; m.p.: 140–142 °C; Yield: 81%; FTIR as KBr disc (cm^−1^): ν(C-H) aromatic 3094, ν(C-H) aliphatic 2966, 2947; ν(C=O) 1712, 1689; ν(C=C) 1549, 1475; ν(C-O) 1275, 1261. ^1^H-NMR (ppm) (CDCl_3_): δ: benzofuran protons 7.32 (t, 7.8 Hz, 1H); 7.50 (t, 7.3 Hz, 1H); 7.58 (d, 8.7 Hz, 1H); 7.62 (s, 1H); 7.72 (d, 7.8 Hz, 1H); benzene protons 6.94 (d, 8.7 Hz, 2H); 8.09 (d, 8.7 Hz, 2H); OCH_3_ 3.87 (s, 3H); CH_2_ 5.51 (s, 2H). ^13^C-NMR (ppm) (CDCl_3_): δ: benzofuran carbons 112.59, 113.55, 123.59, 124.27, 126.82, 128.79, 150.59, 155.73; benzene carbons 113.85, 121.64, 132.23, 163.87; OCH_3_ 55.58, CH_2_ 66.15, COO 165.74, C=O 184.13.

*2‐(1‐Benzofuran‐2‐yl)‐2‐oxoethyl 4‐nitrobenzoate* (**4e**): Solvent for crystal growth: Acetone; m.p.: 180–182 °C; Yield: 84%; FTIR as KBr disc (cm^−1^): ν(C-H) aromatic 3116, ν(C-H) aliphatic 2924, 2858; ν(C=O) 1725, 1694; ν(C=C) 1610, 1475; ν(C-O) 1277, ν(NO_2_) 1524. ^1^H-NMR (ppm) (CDCl_3_): δ: benzofuran protons 7.35 (t, 7.8 Hz, 1H); 7.53 (t, 7.4 Hz, 1H); 7.59 (d, 8.2 Hz, 1H); 7.65 (s, 1H); 7.74 (d, 7.8 Hz, 1H); benzene protons 8.32 (s, 4H); CH_2_ 5.62 (s, 2H). ^13^C-NMR (ppm) (CDCl_3_): δ: benzofuran carbons 112.59, 113.73, 123.67, 124.45, 126.74, 129.04, 150.91, 155.79; benzene carbons 123.75, 131.26, 134.72, 150.35; CH_2_ 66.89, COO 164.23, C=O 183.03.

### 3.4. Antimicrobial Activities

Selected skin diseases microorganisms tested were obtained from the American Type Culture Collection (ATCC). The Gram-positive bacteria used were *Staphylococcus aureus* ATCC 700699, *Streptococcus mutans* ATCC 25175, and *Bacillus licheniformis* ATCC 12759. Four Gram-negative bacteria (*Escherichia coli* ATCC 25922, *Klebsiella pneumoniae* ATCC 13883, *Pseudomonas aeruginosa* ATCC 27853 and *Salmonella typhimurium* ATCC 14028) and one yeast (*Candida albicans* ATCC 10231) were also used for the test.

The minimum inhibition concentration (MIC) of each compound was determined using micro-well dilution method as described by Sivasothy *et al.* [33]. The initial concentrations of each compound ranged from 40–1.25 mg/mL. The lowest concentration which inhibited the growth of the respective bacteria was considered as the MIC. Briefly, 5 µL of compound and 195 µL of bacteria inoculum were added into each well of sterile 96-well plate (Nunc). The final inoculums size was approximately 1.5 × 10^6^ CFU/mL for bacteria and yeast. The antibiotic tetracycline and DMSO (in similar volume with tested compound) were respectively included as positive and negative controls in each plate. The plates were then incubated at 37 °C for 20–22 h. An indicator of bacteria growth, 2-(4-iodophenyl)-3-(4-nitrophenyl)-5-phenyltetrazolium chloride, 95% (INT) (Sigma Aldrich, St Louis, MO, USA) was freshly prepared at initial concentration of 0.3 mg/mL. 40 µL of the solution was added to each well and the plate was further incubated for another 30 min at 37 °C. The assay was performed in triplicate.

### 3.5. Antioxidant Activities

#### 3.5.1. Diphenyl-2-picrylhydrazyl (DPPH) Radical Scavenging Assay

The free radical scavenging activity of each compound was estimated based on DPPH assay as described by Ooi *et al.* [34], with slight modifications. Fifty microliters of the compound (with an initial concentration of 8 mg/mL) was added to 150 µL of ethanolic DPPH solution (300 µM) while ethanol was used as blank. For negative control, 50 µL of DMSO was added to the DPPH solution. The mixture was left to stand for 30 min at 37 °C. Absorbance was read at 515 nm using Multiskan Spectrum microplate reader (Thermo Scientific, Vantaa, Finland). The DPPH scavenging percentage was calculated as follows:

%the DPPH scavenging = [(absorbance of negative control − absorbance of sample)/absorbance of negative control] × 100
(1)

All the experiments were performed in triplicate.

#### 3.5.2. Ferric Reducing Antioxidant Power (FRAP) Assay

The FRAP assay of each compound was carried out based on method described by Ooi *et al.* [34]. The compound (50 μL) at initial concentration of 8 mg/mL was allowed to react with 150 μL of the FRAP solution in a well of a 96-well plate. Triplicate of measurements were performed. The increase in absorbance at 593 nm was measured using a Multiskan Spectrum microplate reader (Thermo Scientific) after 20 min of incubation at 37 °C. DMSO was used as negative control while Trolox (Sigma Aldrich) was used as positive control. The FRAP percentage was calculated as:

%inhibition = [absorbance of sample/maximum absorbance (3.8)] × 100
(2)

#### 3.5.3. Metal Chelating (MC) Assay

The metal chelating assay was performed according to Ooi *et al.* [34]. Briefly, 50 µL of compound (with an initial concentration of 8 mg/mL) was incubated with 5 µL ferrous chloride hexahydrate (2 mM) and 130 µL of deionized water for 5 min. The reaction was initiated by the addition of 15 µL of ferrozine (5 mM). DMSO was prepared as negative control and ethylenediaminetetraacetic acid (EDTA) salt was prepared to be used as positive control. After the mixture has been incubated at room temperature for 10 min, the absorbance was measured at 562 nm using Multiskan Spectrum microplate reader (Thermo Scientific). The metal cheating percentage was calculated using the following equation:

%inhibition = [(absorbance of negative control − absorbance of sample)/absorbance of negative control] × 100%
(2)

All experiments were performed in three replicates.

## 4. Conclusions

A series of five new 2‐(1‐benzofuran‐2‐yl)‐2‐oxoethyl 4-(un/substituted)benzoates **4**(**a**–**e**) was synthesized under mild conditions producing high purity with good yield. The synthesized products **4**(**a**–**e**) are characterized by FTIR, ^1^H-, ^13^C- and ^1^H-^13^C HMQC NMR spectroscopic analysis and their 3D structures were confirmed by single-crystal X-ray diffraction studies. The X-ray diffraction studies revealed the significance of various intermolecular H-bonding interactions contributing to the crystal structure stability. Further, the compounds were screened for their antibacterial (four gram negative and three gram positive bacterial strains) and antifungal (*Candida albicans*) activities using tetracycline as standard. The results indicated the tested compounds possess antimicrobial ability in the order of **4b** < **4a** < **4c** < **4d** < **4e**. The highest potency with MIC value of 125 µg/mL was observed for compound **4e**. The results of antioxidant activities revealed the highest activity for diphenyl-2-picrylhydrazyl (DPPH) radical scavenging, ferric reducing antioxidant power (FRAP) assay and metal chelating (MC) activity are found in **4e** (32.62% ± 1.34%), **4d** (31.01% ± 4.35%) and **4a** (27.11% ± 1.06%), respectively. In conclusion, among the synthesized benzofuranyl esters, compound **4e** may be a potential antimicrobial and antioxidant agent. The results of the bioassay revealed the vital effect of substituent change on antimicrobial and antioxidant activities.

## Supplementary Materials

Supplementary materials can be accessed at: http://www.mdpi.com/1420-3049/20/09/16566/s1.
