# Supplementary material for: Benzofuranyl Esters: Synthesis, Crystal Structure Determination, Antimicrobial and Antioxidant Activities
Source: Molecules. 2015 Sep 11;20(9):16566–81. doi: 10.3390/molecules200916566 (PMC6332186; doi:10.3390/molecules200916566)
Supplement: Supplementary file 1 [file molecules-20-16566-s001.pdf]

Supplementary Information

Additional Part for X-ray Crystal Structure Description

C–H···π interactions are observed between two inversion-related molecules in **4a**, involving the centroids of five-membered and six-membered rings of benzofuran moiety as shown in Figure S1. In **4b**, the zigzag chains are stacked along *b*-axis (Figure S2) under weak π···π interactions (3.9162 (16) Å; −*x*, −*y*, −*z*) in between O1*A*/C1*A*/C6*A*/C7*A*/C8*A* and C12*A*–C17*A* rings. Two C–H···π interactions are observed in the crystal of **4c** (Figure S3). One of the interactions is formed in between two parallel-displaced phenyl ring whereas the other one formed edge-to-face interaction. Two π···π interactions are observed in between two benzofuran rings with equal centroid-to-centroid distance of 3.6111 (4) Å (symmetry code −*x* + 1, −*y* + 1, −*z*). For **4d**, π···π interactions are observed in between two benzofuran rings (centroid-to-centroid distance = 3.5820 (3) Å, −*x*, −*y* + 2, −*z*) which are similar to **4c**. Two C–H···π interactions (Figure S4) are formed in between the sheets which further consolidate the interaction pattern into a three-dimensional network. The molecules in **4e** are linked by weak C–H···O hydrogen bonds (Table S1) into two-dimensional sheets parallel to *ac*-plane. Figure S5a,b show sheets *A* and *B* which are fully comprised of molecule *A* or molecule *B*, respectively. Weak C–H···O and π···π interactions (Table S2) link the hydrogen bonded sheets in …*AA_invBB_inv*… pattern along *b*-direction into a three-dimensional network (Figure S6). Sheet *A_inv* is an inversion of sheet *A* and the same applies to *B_inv*.

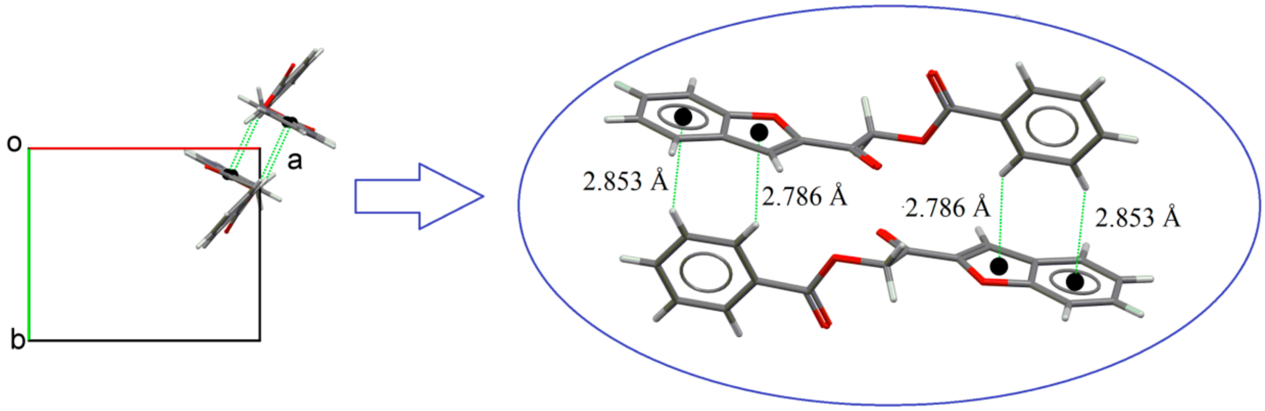

Figure S1. C–H···π interactions (green dotted lines) are observed in between two inversion-related molecules of **4a** with H···centroid distances of 2.79 Å and 2.85 Å.

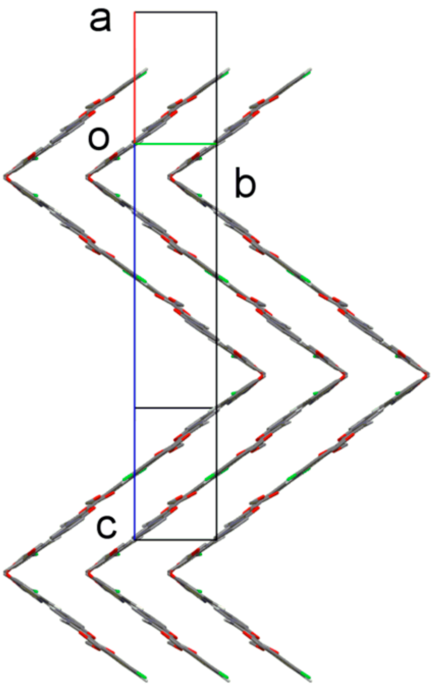

Figure S2. Partial packing diagram of **4b** shows three zigzag chains viewed along *a*-axis.

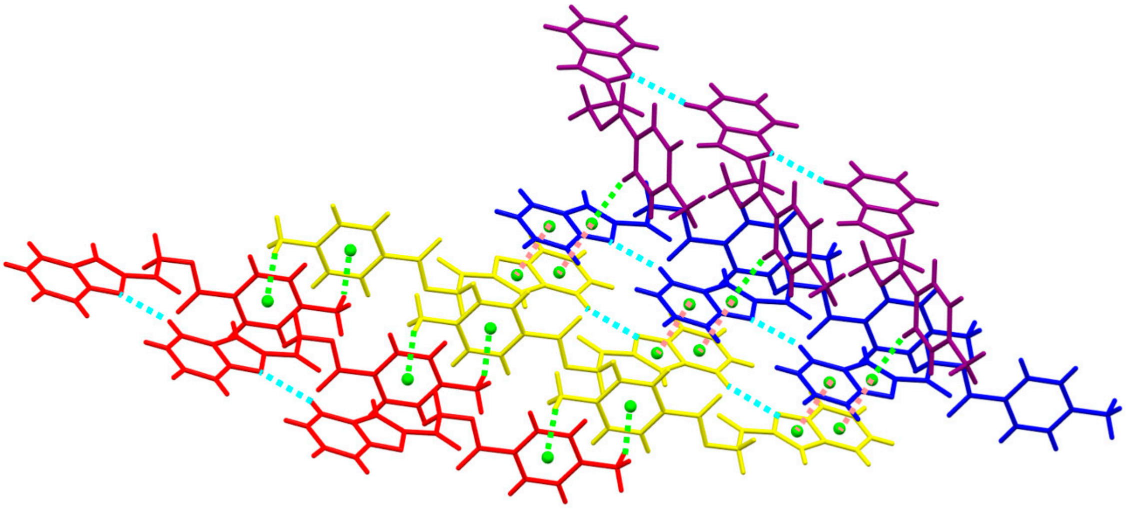

Figure S3. C–H···O hydrogen bonds (cyan dotted lines), C–H···π (green dotted lines) and π···π (pink dotted lines) interactions contribute to the formation of the three-dimensional network in **4c**. Each hydrogen-bonded chain is drawn in different colours.

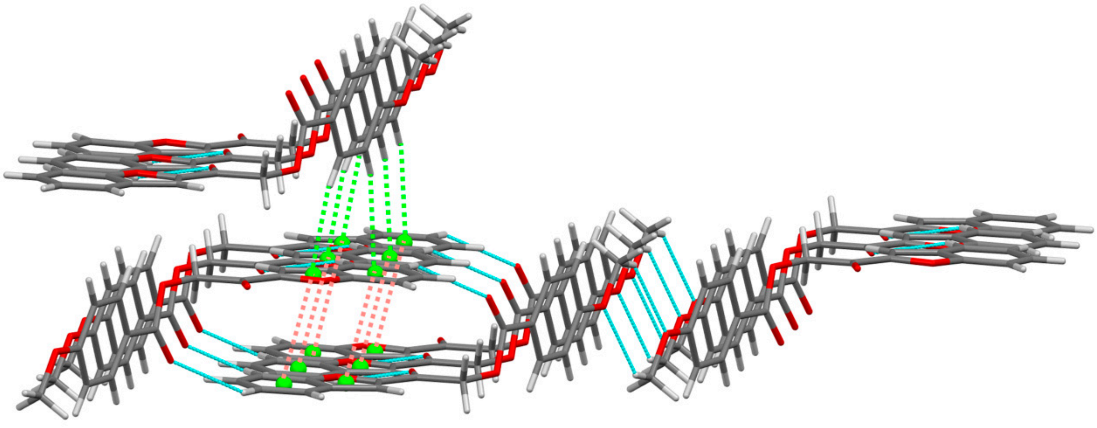

Figure S4. Cyan dotted lines show C–H···O hydrogen bond whereas green and pink dotted lines show C–H···π and π···π interactions, respectively in **4d**.

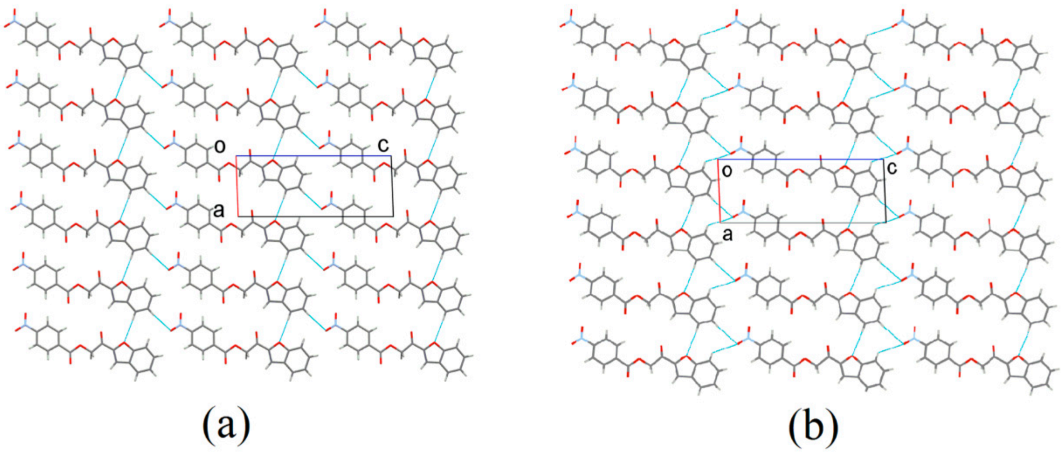

Figure S5. C–H···O hydrogen bond interactions (blue dotted lines) form two-dimensional sheets *A* (**a**) and *B* (**b**) parallel to *ac*-plane.

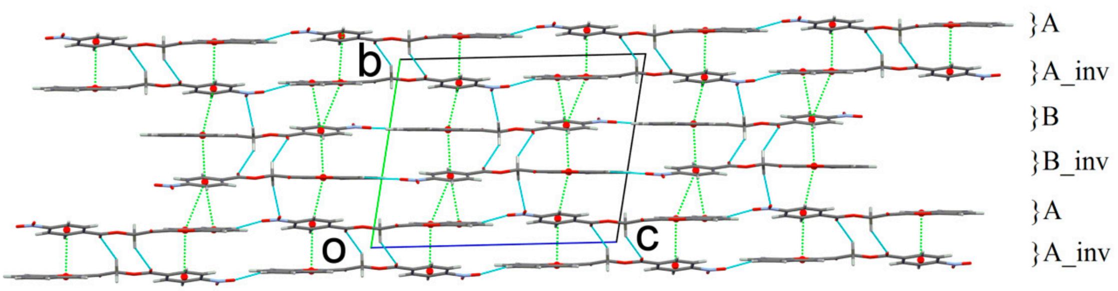

Figure S6. Three-dimensional view of C–H···O hydrogen bonds (blue dotted lines) and π···π interactions (green dotted lines) in **4e**.

Table S1. Hydrogen bond geometries for **4**(**a**–**e**).

D–H···*A*	*D*–H (Å)	H···*A* (Å)	*D*···*A* (Å)	*D*–H···*A* (°)	Symmetry Code
**4a**					
C4–H4*A*···O4	0.93	2.54	3.368 (2)	148	$-x + 3/2, y - 1/2, -z + 1/2$
C10–H10*A*···O4	0.97	2.60	3.445 (2)	145	$x + 2, -y + 1, -z$
C14–H14*A*···O3	0.93	2.69	3.312 (3)	125	$-x + 3/2, y + 1/2, -z - 1/2$
C16–H16*A*···Cg2	0.93	2.85	3.6715 (19)	147	$-x + 2, -y, -z$
C17–H17*A*···Cg1	0.93	2.79	3.5489 (17)	140	$-x + 2, -y, -z$
**4b**					
C5*B*–H5*B*···O4*A*	0.93	2.58	3.347 (11)	139	$x, y + 1, -z + 1/2$
C7*A*–H7*A*···O3*B*	0.93	2.40	3.18 (2)	141	$-x, y - 1, -z + 1/2$
C14*B*–H14*B*···O4*A*	0.93	2.52	3.237 (12)	134	$x, y + 1, z$
C16*A*–H16*A*···O1*A*	0.93	2.54	3.473 (3)	175	$-x, -y + 1, -z$
**4c**					
C5–H5*A*···O1	0.93	2.51	3.3903 (17)	158	$x, y - 1, z$
C17–H17*A*···Cg1	0.93	2.68	3.5571 (17)	157	$-x + 3/2, y + 1/2, -z + 1/2$
C18–H18*B*···Cg3	0.96	2.78	3.7180 (19)	167	$-x + 1, -y + 3, -z + 1$
**4d**					
C4–H4*A*···O4	0.93	2.56	3.3026 (18)	137	$-x, -y + 2, -z$
C5–H5*A*···O3	0.93	2.53	3.3100 (18)	141	$x - 1, y, z$
C18–H18*A*···O5	0.96	2.59	3.321 (2)	133	$-x + 3, -y + 1, -z + 1$
C16–H16*A*···Cg2	0.93	2.93	3.7004 (16)	141	$-x + 1, -y + 1, -z$
C17–H17*A*···Cg1	0.93	2.82	3.6435 (14)	148	$-x + 1, -y + 1, -z$
**4e**					
C4*A*–H4*AA*···O6*A*	0.93	2.70	3.619 (5)	169	$x + 1, y, z + 1$
C5*A*–H5*AA*···O1*A*	0.93	2.62	3.543 (4)	172	$x + 1, y, z$
C10*A*–H10*B*···O4*A*	0.97	2.64	3.488 (4)	146	$-x + 1, -y, -z$
C2*B*–H2*BA*···O5*B*	0.93	2.69	3.318 (4)	125	$x, y, z - 1$
C4*B*–H4*BA*···O5*B*	0.93	2.66	3.583 (5)	174	$x - 1, y, z - 1$
C5*B*–H5*BA*···O1*B*	0.93	2.62	3.548 (4)	174	$x - 1, y, z$
C10*B*–H10*D*···O4*B*	0.97	2.68	3.532 (5)	146	$-x + 1, -y + 1, -z + 1$

Cg1: O1/C1/C6/C7/C8, Cg2: C1–C6, Cg3: C12–C17.

Table S2. π···π interactions in **4**(**b**–**e**).

Centroid 1	Centroid 2	Centroid-to-Centroid Distance (Å)	Symmetry Code
**4b**			
Cg1	Cg2	3.9162 (16)	$-x, -y, -z$
**4c**			
Cg3	Cg4	3.6111 (10)	$-x + 1, -y + 1, -z$
**4d**			
Cg3	Cg4	3.5820 (8)	$-x, -y + 2, -z$
**4e**			
Cg1	Cg2	3.7263 (19)	$-x, -y, -z$
Cg1	Cg7	3.7794 (19)	$-x + 1, -y + 1, -z + 1$
Cg5	Cg7	3.8710 (2)	$-x + 1, -y + 1, -z + 1$
Cg2	Cg6	3.9167 (19)	$-x + 1, -y + 1, -z$
Cg6	Cg7	3.7022 (19)	$-x + 2, -y + 1, -z + 1$

Cg1, Cg2, Cg3, Cg4, Cg5, Cg6 and Cg7 are the centroids of O1*A*/C1*A*/C6*A*/C7*A*/C8*A*, C12*A*–C17*A*, O1/C1/C6/C7/C8, C12–C17, C1*A*–C6*A*, O1*B*/C1*B*/C6*B*/C7*B*/C8*B* and C12*B*–C17*B* rings, respectively.
